# Acute intrathoracic gastric herniation as a rare cause of cardiac arrest

**DOI:** 10.1186/cc12238

**Published:** 2013-03-19

**Authors:** DW Hoelen, AL Van Duijn, CL Meuwese, JP Ruurda, MA Sikma

**Affiliations:** 1 UMC Utrecht, the Netherlands

## Introduction

In this case report, we describe a patient who presented with a cardiac arrest as a result of an obstructive shock, which progressed into cardiac arrest, caused by an acute para-esophageal gastric herniation.

## Methods

Our patient, with a medical history of a laparoscopic repair of a symptomatic diaphragmatic hernia 6 months prior, presented herself at the emergency department with pain in the upper abdomen and nausea. The physical examination, laboratory tests and X-ray of the thorax were normal and she was sent home. Twenty-four hours later paramedics were summoned to our patient because of increased complaints. On arrival of the paramedics she had a normal electrocardiogram (ECG) and during the transfer from her bed to the stretcher she collapsed due to pulseless electric activity (PEA), for which cardiopulmonary resuscitation was started. Sinus rhythm and output was regained after several minutes and the patient was transported to the hospital. At arrival in the hospital, the X-ray of the thorax showed an intrathoracic stomach and a significant mediastinal shift to the right.

## Results

After emergency laparotomy, which concerned correcting the gastric herniation and resection of an ischemic part of stomach, the patient remained hemodynamically stable. Cardiac ischemia was ruled out based on ECG, laboratory findings, cardiac ultrasound and cardiac computed tomography. The ultrasound in the emergency department did show a distended right ventricle and normal left function, which disappeared later (after repositioning the stomach), which is evidence for the mediastinal shift as a cause for the PEA.

## Conclusion

We are the first to describe a patient requiring cardiopulmonary resuscitation for progressive obstructive shock, due to an intrathoracic stomach. Especially after a laparoscopic repair of a diaphragmatic hernia, this is a rare cause for shock and cardiac arrest, which requires a different medical approach.

